# Allele-Specific Disruption of a Common *STAT3* Autosomal Dominant Allele Is Not Sufficient to Restore Downstream Signaling in Patient-Derived T Cells

**DOI:** 10.3390/genes13101912

**Published:** 2022-10-20

**Authors:** Saskia König, Manfred Fliegauf, Manuel Rhiel, Bodo Grimbacher, Tatjana I. Cornu, Toni Cathomen, Claudio Mussolino

**Affiliations:** 1 Institute for Transfusion Medicine and Gene Therapy, Medical Center–University of Freiburg, Breisacherstr. 115, 79106 Freiburg, Germany; 2 Center for Chronic Immunodeficiency, Medical Center-University of Freiburg, Breisacherstr. 115, 79106 Freiburg, Germany; 3 Institute for Immunodeficiency, Medical Center-University of Freiburg, Breisacherstr. 115, 79106 Freiburg, Germany; 4 CIBSS-Centre for Integrative Biological Signaling Studies, University of Freiburg, 79106 Freiburg, Germany; 5 Faculty of Medicine, University of Freiburg, 79106 Freiburg, Germany; 6 RESIST-Cluster of Excellence 2155 to Hannover Medical School, Satellite Center Freiburg, Freiburg, Germany; 7 DZIF-German Center for Infection Research, Satellite Center Freiburg, Freiburg, Germany

**Keywords:** allele-specific nuclease, gene therapy, CRISPR-Cas, genome editing, primary immunodeficiency

## Abstract

Dominant negative mutations in the *STAT3* gene account for autosomal dominant hyper-IgE syndrome (AD-HIES). Patients typically present high IgE serum levels, recurrent infections, and soft tissue abnormalities. While current therapies focus on alleviating the symptoms, hematopoietic stem cell transplantation (HSCT) has recently been proposed as a strategy to treat the immunological defect and stabilize the disease, especially in cases with severe lung infections. However, because of the potentially severe side effects associated with allogeneic HSCT, this has been considered only for a few patients. Autologous HSCT represents a safer alternative but it requires the removal of the dominant negative mutation in the patients’ cells prior to transplantation. Here, we developed allele-specific CRISPR-Cas9 nucleases to selectively disrupt five of the most common *STAT3* dominant negative alleles. When tested ex vivo in patient-derived hematopoietic cells, allele-specific disruption frequencies varied in an allele-dependent fashion and reached up to 62% of alleles harboring the V637M mutation without detectable alterations in the healthy *STAT3* allele. However, assessment of the gene expression profiles of the STAT3 downstream target genes revealed that, upon activation of those edited patient cells, mono-allelic STAT3 expression (functional haploinsufficiency) is not able to sufficiently restore STAT3-dependent signaling in edited T cells cultured in vitro. Moreover, the stochastic mutagenesis induced by the repair of the nuclease-induced DNA break could further contribute to dominant negative effects. In summary, our results advocate for precise genome editing strategies rather than allele-specific gene disruption to correct the underlying mutations in AD-HIES.

## 1. Introduction

Signal transducer and activator of transcription 3 (STAT3) is a key cytoplasmic transcription factors that regulates immune cell activation and differentiation through the transduction of signals initiated by a wide variety of cytokines and growth factors [1]. Given its critical role in immune homeostasis [2] and the regulation of both innate and adaptive immunity [3], pathogenic variants in the *STAT3* gene account for a variety of human conditions including immune deficiencies and cancer [4]. In particular, heterozygous autosomal dominant (AD) mutations in *STAT3* have been associated with hyper-immunoglobulin E syndrome (HIES), a rare primary immune deficiency characterized by elevated serum IgE levels and recurrent skin and lung infections [5]. Since the description of the first *STAT3* missense mutation associated with AD-HIES [6], more than 140 variants have been identified, largely expected to exert a DN effect [7]. Since STAT3 functions as a dimer, the incorporation of the mutated STAT3 within the dimer generally impairs its function. As a consequence, mutated STAT3 has a dominant negative effect on the remaining normal STAT3 protein and this results in the failure of naïve T cells to differentiate into Th17 cells, with a subsequent failure of IL-17 and IL-22 secretion [8]. STAT3-HIES is further associated with multi-organ dysfunction, which begins during infancy and leads to high morbidity and mortality with an average life expectancy of less than 10 years if left untreated [9]. Treatment is typically prophylactic in order to prevent bacterial and fungal infections, but this rarely prevents lung, oral mucosa, and skin involvement, leads to drug resistance, and is not curative. Since STAT3-HIES is a primary immunodeficiency, it could, in principle, be treated by allogeneic hematopoietic stem cell transplantation (HSCT). While two initial reports of failure have strongly discouraged this approach [10], data on more recently transplanted individuals call for cautious optimism due to their significant stabilization of most disease parameters including disabling lung disease [11,12]. The intrinsic risks of HSCT for the development of life-threatening complications such as graft-versus-host disease or vascular anomalies [13] have to be carefully considered, especially in STAT3-HIES patients that are often not at immediate risk of death but suffer from an ever-decreasing quality of life as they age. Autologous HSCT mitigates some of the risks associated with the HSCT procedure but it is meaningful only when combined with the correction of the disease-underlying mutations in the patient-derived cells. Since evidence from mouse models has shown that animals with a complete deletion of a single *STAT3* allele are phenotypically normal [14], strategies that either correct or eliminate the mutated dominant negative *STAT3* allele might be beneficial for STAT3-HIES patients. The latter could be achieved with designer nucleases, engineered proteins capable of introducing a DNA double strand break (DSB) in the DNA helix at desired genomic locations [15]. This event results in the activation of cellular DNA repair mechanism, mainly non-homologous end-joining (NHEJ) or homology-directed repair (HDR), which repairs the DNA break to maintain genome integrity. However, the longer-term presence of the nuclease results in consecutive DSB formation until insertion or deletion (indel) mutations occur, which eventually inhibit its activity [16]. Therefore, out-of-frame indels occur on average in two thirds of cases, resulting in target gene inactivation. The introduction of indel mutations into the coding regions of genes has been explored in the last decade to develop therapies for a variety of human disorders, with the first clinical results recently published [17,18]. Considering the steps forward that precision medicine has made since its inception, new opportunities can also be envisioned for treating STAT3-HIES.

Here, we explored the high-specificity of the CRISPR-Cas9 system to selectively disrupt, in an allele-specific manner, the *STAT3* allele harboring the autosomal dominant mutation causing STAT3-HIES. We selected five common dominant-negative *STAT3* mutations in an attempt to validate a strategy that would be applicable to the majority of STAT3-HIES patients. We successfully developed allele-specific nucleases capable of inactivating the selected mutated alleles through the deposition of indel mutations with frequencies approaching 62%, without any effect on the healthy allele. However, when tested functionally, the edited cells were indistinguishable from non-edited cells, suggesting that STAT3 expression driven from a single allele is not sufficient to restore proper STAT3 signaling. 

## 2. Materials and Methods

### 2.1. Plasmids

**Figure 1:**
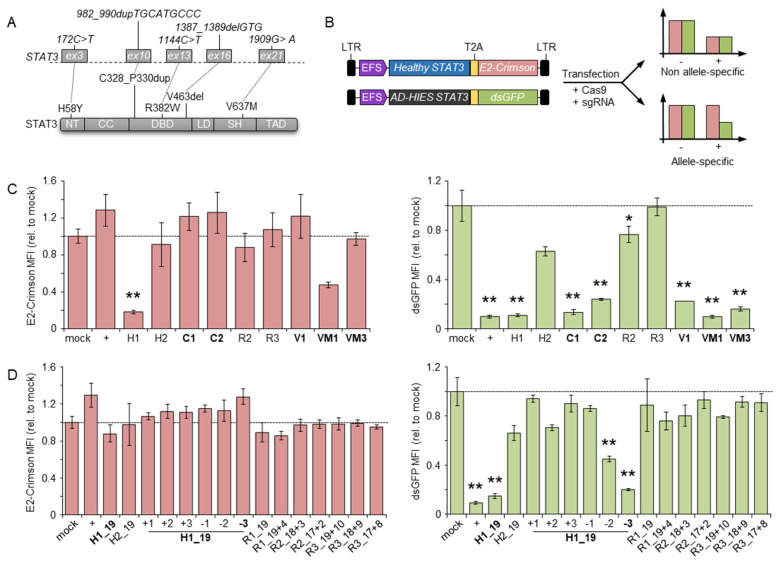
Identification of allele-specific nucleases. (**A**) Schematics of the *STAT3* gene (top) and the corresponding protein domain structure (bottom). The selected mutations at both the DNA and protein levels are highlighted. The *STAT3* exons harboring the selected mutations are indicated with grey rectangles. (**B**) Schematics of the two reporter constructs (left) and principle of the reporter assay. After transfection, the expression level of the marker genes is indicative of the ability of the designer nucleases to discriminate between the healthy vs. the mutated (AD-HIES) STAT3 variant. LTR: long terminal repeat; T2A: 2A self-cleaving peptide. (**C**,**D**) Marker gene expression levels. The bar graphs show the mean fluorescence intensity (MFI) of either E2-Crimson (left) or dsGFP (right), respectively, determined by flow cytometry of the transfected cell population (expressing a blue fluorescent protein, not indicated). CRISPR-Cas9 with different designs were used including full-length (**C**) or truncated and mismatched (**D**) single guide RNAs named as shown in Table 1. The data summarized at least three biological replicates. Statistically significant differences compared to cells transfected with a mock nuclease (targeting the CCR5 gene) are indicated with an asterisk and the corresponding *p*-values were calculated with one-way ANOVA followed by Dunnett’s multiple comparisons test (* *p* < 0.05, ** *p* < 0.01). Error bars indicate s.e.m. Allele-specific nucleases are highlighted in bold. NT: N-terminal domain; CC: coiled-coil domain; DBD: DNA binding domain; LD: linker domain; SH: Src Homology 2 domain; TAD: transcriptional activation domain; +: nuclease targeting the *GFP* gene.

The expression plasmid for the *Streptococcus pyogenes* Cas9 was kindly provided by J. Keith Joung (Addgene plasmid #43861). All of the single guide RNA (sgRNA) expression plasmids were cloned through an oligo-cloning procedure. In brief two complementary oligonucleotides (ODN, purchased at Integrated DNA Technology; Table S1) were resuspended to 100 μM in distilled water and 1 μL of each ODN was mixed with 10 μL of annealing buffer (100 mM Tris/HCl pH 7.5, 500 mM NaCl) and 88 μL of nuclease-free water. After incubation for 10 min at 95 °C, the reaction was slowly cooled down to room temperature. All the ODNs, after annealing, resulted in a dsODN with 5′-ACAC and 3′-AAAA overhangs. The dsODN was then ligated into the sgRNA expression vector MLM3636 (kind gift from J. Keith Joung, Addgene plasmid #43860) digested with the *BsmB*I restriction enzyme in a 5:1 molar ratio. The fluorescent reporter constructs were generated from a pCCL third generation lentiviral mammalian expression vector (laboratory ID #890). First, a multiple cloning site was introduced into the plasmid #890 digested with *Sal*I and *Nhe*I by oligo-cloning of the primers #2413 (5′-ctagccgacctgcaggtaccggtggatccatgcatg) and #2414 (5′-tcgacatgcatggatccaccggtacctgcaggtcgg), as described above, resulting in plasmid #890-MCS. Subsequently, the open reading frame (ORF) of a destabilized green fluorescence protein (*dsGFP*), which included a self-cleaving T2A peptide, was released from the plasmid #1265 via *BamH*I and *Age*I digestion and ligated using the same restriction sites into the #890-MCS, resulting in #890-MCS-T2A-dsGFP. A gBlock (Integrated DNA Technology) containing the sequence of the EF1α short promoter (EFS) and the *STAT3* ORF including the five selected mutations (Figure 1A) was cloned via the Gibson Assembly upstream of the T2A in the #890-MCS-T2A-dsGFP linearized with *Nhe*I and *Sfb*I, resulting in plasmid #1664 (pCCL_EFS_STAT3mut_T2A_dsGFP). To generate the reporter plasmid #1665 (pCCL_EFS_STAT3wt_T2A_E2Crimson), the mutated *STAT3* and the dsGFP sequences from #1664 were replaced via Gibson Assembly using two gBlocks (IDT) containing either the normal *STAT3* ORF (using *Nhe*I and *Sfb*I restriction enzymes) or the *E2-Crimson* ORF (using *Sbf*I and *Sal*I restriction enzymes), respectively.

### 2.2. Cell Culture 

The cell lines described in this study were authenticated using a 16 DNA marker profile (Eurofins Genomics, Tokyo, Japan). HEK293T cells and derivate reporters were kept in Dulbecco’s modified Eagle medium (Life Technologies, Carlsbad, CA, USA) supplemented with 10% FCS (Thermo Fisher Scientific, Waltham, MA, USA), 1% penicillin/streptomycin (Merck, Kenilworth, NJ, USA), 1 mM sodium pyruvate (Merck), and grown in plates for adherent cells (Sarstedt, Nümbrecht, Germany) kept at 37 °C in 5% CO_2_. 

Human peripheral blood mononuclear cells (PBMCs) were isolated from leukocyte reduction system (LRS) chambers by density gradient centrifugation (Ficoll-Paque) and kindly provided by the Blood Donation Center of the Medical Center, University of Freiburg after routine plateletpheresis and anonymized informed donor consent. PBMCs from AD-HIES patients were purified from whole blood samples received from the FREEZE Biobank of the Medical Center–University of Freiburg after anonymizing the informed donor consent and approval of the local ethics committee. In this case, PBMCs were isolated and purified on a Biocoll gradient (Biochrom, Germany) and used for allele-specific knock out experiments. PBMCs were kept in RPMI 1640 medium (Life Technologies) supplemented with 10% FCS (Thermo Fisher Scientific), 1% penicillin/streptomycin (Merck), and 100 U/mL IL-2 in plates for suspension cells (Sarstedt) at 37 °C in 5% CO_2_ for 4 h before activation with the T Cell Activation/Expansion Kit (Miltenyi) according to the manufacturer’s protocol. After 3 days, beads were removed and the cells were expanded at a concentration of 1.0 × 10^6^ cells/mL or 0.5 × 10^6^ cells/cm². When necessary, the cell number and viability were measured using the NucleoCounter NC-250 (ChemoMetec) and Solution 18 containing acridine orange and DAPI according to the manufacturer’s protocol. For genome editing experiments, 1.0 × 10^6^ PBMCs were transfected four days after thawing (or one day after beads removal). To this end, 20 pmol Cas9 protein (Integrated DNA Technology) and 100 pmol of the indicated sgRNAs (Synthego; the sgRNA included two phosphorothioate modifications at both the 5′- and 3′-ends) were precomplexed at 37 °C for 10 min before transfection using the P3 Primary Cell 4D-Nucleofector (Lonza) and the EO115 program. Afterward, cells were seeded in a 48-well plate and expanded until further analysis. For functional assays, IL2 was removed one day before stimulating 0.5–1.0 × 10^6^ the PBMCs for 1 h with 50 ng/mL IL21 (PeproTech).

### 2.3. STAT3 Reporter Assay

Allele-specific nucleases were preselected in an episomal reporter assay. One day before transfection, 100′000 HEK293T cells were seeded in a 24-well plate in 450 μL medium. The day of transfection, the cells were transfected with a DNA mixture containing 100 ng of each reporter plasmid, 600 ng of Cas9, and 200 ng of sgRNA expression plasmids, respectively. To normalize for transfection efficiency, the DNA mixture contained 50 ng of a BFP expression plasmid to reach a total amount of 1250 ng of transfected DNA. Two days after transfection, the cells were analyzed by flow cytometry on a BD FACSCanto II (Becton Dickinson). After isolating the single (FSC-A/FSC-H) and living cells (FSC-A/SSC-A), the mean fluorescence intensity (MFI) of dsGFP and E2-Crimson were recorded in the BFP-positive cell population. All samples were analyzed in technical triplicates and the assay included at least three biological replicates. For allele-specificity validation in the HEK-STAT3mut cells, 200,000 cells were nucleofected using the CM130 program using the SF Cell Line 4D-Nucleofector (Lonza). The nucleofection mix included the Cas9 protein and the selected sgRNA, preassembled to form active RNPs at 37 °C for 10 min in a 1:1 molar ratio with 30 pmol each. Nucleofected cells were seeded in a 24-well plate and expanded until further analysis.

### 2.4. Next Generation Sequencing

Allele specificity of the selected nucleases in patient-derived PBMCs was assessed via next generation sequencing. At the day of analysis, cells were harvested and genomic DNA isolated using the NucleoSpin Tissue gDNA Extraction Kit (Macherey Nagel) following the manufacture’s procedure. PCR amplicons encompassing the nuclease target sites were amplified from 50 ng of genomic DNA using primers specific for each *STAT3* mutation (Table S1) using Q5 Polymerase (New England Biolabs) and an annealing temperature of 69 °C with 35 cycles. The PCR amplicons were purified using the QIAquick PCR Purification Kit (Qiagen) and resuspended in 30 μL of nuclease-free water. The NGS library was prepared from 20 ng of purified PCR products using the NEBNext Ultra II DNA Library Prep Kit and NEBNext Multiplex Oligos for Illumina (New England Biolabs) following the manufacturer’s recommendations. To this end, the adaptors were diluted 1:10 in TE buffer as suggested. The library was then quantified using the ddPCR Library Quantification Kit for Illumina TruSeq (Bio-Rad Laboratories) and the QX200 Droplet Digital PCR System (Bio-Rad Laboratories) following the manufacturer’s instructions. The libraries were then sequenced using the MiSeq Reagent Kit v2 and the MiSeq sequencing system (Illumina) according the manufacturer’s guidelines. The data analysis was performed using the CRISPResso2 online tool [19]. The number of healthy (non-edited) *STAT3* reads or alleles harboring either the selected *STAT3* mutation or newly formed indels was calculated as the percentage fraction of the total aligned reads.

### 2.5. Functional Evaluation of STAT3 Signaling 

The effects of the STAT3-HIES allele-specific inactivation on STAT3 signaling were assessed via analysis of the expression levels of STAT3-specific transcriptional target genes by droplet digital PCR. At the indicated time points, the total RNA was extracted utilizing the RNeasy Mini Kit (Qiagen) following the manufacturer’s protocol and the RNA concentration and quality were determined using the Nanodrop 2000 device (Thermo Fisher Scientific). A total of 150 ng of RNA was reverse transcribed using the QuantiTect Reverse Transcription Kit (Qiagen) following the manufacturer’s recommendations. The expression levels of selected STAT3-specific transcriptional targets were measured using the QX200 Droplet Digital PCR System (Bio-Rad Laboratories) and the QX200 ddPCR EvaGreen Supermix. The primers used to detect *HPRT1*, *SOCS3*, *BATF*, and *IRF4* expression are listed in Table S1. Data were normalized to the levels of the endogenous *HPRT1* gene.

## 3. Results

### 3.1. Selection of Designer Nucleases Specific for the Dominant Negative STAT3 Alleles

To establish a targeting strategy that could be applicable to the majority of STAT3-HIES patients, we selected five frequent *STAT3* mutations that accounted for 47% of the total patients identified in our cohort [20]. These included three mutations of different types affecting the DNA binding domain, namely the point mutation R382W (c.1144C > T), the deletion V463del (c.1387_1389delGTG), and the three amino acid duplications C328_P330dup (c.982_990dupTGCATGCCC). In addition, we selected a mutation commonly found in the STAT3 coiled-coil domain (H58Y, c.172C > T), and finally, the V637M (c.1909G > A) in the SH2 domain (Figure 1A). Notably, R382W and V637M were the most frequent DN variants in STAT3 HIES with a frequency of 24% and 15% in our patient cohort, respectively [20].

We used the CRISPR-Cas9 genome editing system to introduce a DSB in proximity to the selected mutations. Targeted indel formation, consequent to the repair of the Cas9-induced DSB via NHEJ, eventually led to allele-specific inactivation. Given the high sequence identity between the two *STAT3* alleles, we exploited various ways to favor the targeting of the mutated *STAT3* allele. Since mismatches in the ‘seed sequence’ of the CRISPR-Cas protospacer have a stronger impact on Cas9 binding [21], we first identified target sequences that placed the disease-underlying mutation as close as possible to the protospacer adjacent motif (PAM; Table 1).

We reasoned that such a design would impair binding of the CRISPR-Cas9 nuclease to the healthy *STAT3* allele, thus favoring the occurrence of DSBs in the mutated allele. To facilitate the screening of CRISPR-Cas9 nucleases capable of allele-specific targeting, we established a dual fluorescent reporter system. A first expression plasmid encoded a fusion protein that included the red fluorescent E2-Crimson protein fused by a T2A self-cleaving peptide to the normal STAT3. A second plasmid coded for a destabilized green fluorescent protein (dsGFP) fused to an artificial STAT3 harboring the five selected mutations. We then assessed the activity of the designer nucleases by co-transfection of their respective expression plasmids with the reporter plasmids in a HEK293T cell line. A reduction in the mean fluorescent intensity (MFI) of either E2-Crimson or dsGFP signals compared to the controls is therefore indicative of the nuclease ability to cleave the respective plasmid. In particular, reduction in the green signal with the absence of red signal alterations identifies an allele-specific nuclease (Figure 1B). We used this system to assess the ability of the CRISPR-Cas9 system to discriminate between the two *STAT3* alleles and included a third plasmid expressing a blue fluorescent protein (BFP) to normalize for transfection efficiency (Figure S1). Using this reporter system, we identified allele-specific CRISPR-Cas9 nucleases for targeting three out of five selected *STAT3* mutations, namely, the C1 and C2 for the C328_P330dup, the V1 to inactivate the V463del allele, and VM1 and VM3 targeting the V637M mutation, respectively (Figure 1C). For the remaining H58Y and R382W alleles, we were unable to design allele-specific CRISPR-Cas9 nucleases with this strategy. To enhance the ability of the CRISPR-Cas9 complex to discriminate between the two highly similar *STAT3* target sites, we shortened the 5′-end of the respective guide RNA to 17, 18, and 19 nucleotides (Table 1). As shown previously [22], such a design reduces the binding energy of the CRISPR-Cas9 complex to a level just sufficient for binding and cleaving a fully matched target site but is more sensitive to even a single base mismatch. We combined this strategy with the introduction of additional mismatched nucleotides flanking the *STAT3* mutation in the gRNA to further destabilize the binding to the non-target allele (Table 1). Testing these CRISPR-Cas9 nuclease variants in the reporter system revealed that shortening of the H58Y-targeting gRNA resulted in the identification of an allele-specific nuclease that retained high activity on the mutant allele and lost its ability to disrupt the normal *STAT3* reporter (H1_19, Figure 1D). The addition of further mismatched nucleotides in the truncated H1 gRNA largely abolished nuclease activity, except for two cases, with one of the two being highly specific (H1_19–3, Figure 1D). On the other hand, allele-specific targeting of the R382W allele failed with all of the tested strategies (Figure 1D), even though a PCR amplicon containing the R382W target was efficiently cleaved in vitro by the corresponding ribonucleoprotein (RNP) complex (Figure S2).

### 3.2. Validation of Allele-Specificity at Genomic Sites

**Figure 2:**
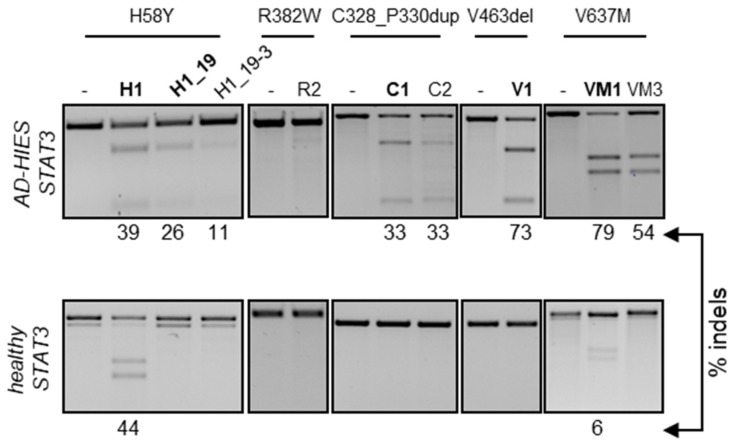
Chromosomal activity of allele-specific nucleases. The HEK293T reporter cells were nucleofected with the indicated nucleases in the form of preassembled RNP or left untreated (-). Three days later, genomic DNA was extracted and subjected to PCR amplification of the target loci harboring the indicated *STAT3* mutation (top). The different amplicons, either derived from the integrated reporter construct (top images) or from the endogenous *STAT3* alleles (bottom images) were assessed for the presence of nuclease-induced indels by the T7E1 assay. The percentage of cleaved product is indicated below each panel. Nucleases selected for experiments in primary cells are highlighted in bold.

Having identified at least one allele-specific nuclease for four out of the five *STAT3*-HIES alleles, we sought to validate their activity in the genomic context. We hypothesized that episomal targeting of the fluorescent reporters might be facilitated by the absence of chromatin and targeting efficiency might be negatively affected in the context of the target genome. To this end, we generated a HEK293T reporter cell line stably expressing the dsGFP-reporter containing the *STAT3* allele harboring the five selected STAT3-HIES mutations (HEK-STAT3mut) via lentiviral transduction, as previously described [23]. Upon clonal expansion, we measured the copy number of the integrated reporter via droplet digital PCR. As a control, the copy numbers of a reference gene (*PTBP2*) and of *STAT3* were measured in human-derived peripheral blood mononuclear cells (PBMCs) and the parental HEK293T cells. While PBMCs harbored two copies of each gene, hypotriploid HEK293T cells had four copies of *STAT3* and three copies of *PTBP2*, respectively (Table S2). We performed the same analysis in two HEK-STAT3mut clones and confirmed the presence of four *STAT3* copies while the copy number of the integrated reporter ranged from two to three, respectively (Table S2). We used HEK-STAT3mut clone #7 to validate the ability of the selected nucleases to discriminate between the endogenous healthy *STAT3* alleles and the integrated reporter harboring the selected *STAT3* pathogenic variants. To this end, HEK-STAT3mut clone #7 cells were nucleofected with preassembled RNPs that included the Cas9 protein and either of the most efficient gRNAs selected with the episomal reporter. To monitor the occurrence of indel mutations, we employed the T7 endonuclease 1 (T7E1) assay, as previously described [24]. In line with the episomal results, all of the selected nucleases successfully targeted the integrated reporter with efficiencies ranging from 30% to 70% (Figure 2). Interestingly, the episomal reporter assay was able to predict subtle differences in targeting efficiencies as well as in the ability to discriminate between the healthy and mutant alleles, which were confirmed on the genome level. Indeed, in both assays, VM3 was not active on the healthy allele compared to VM1. Similarly, VM1 was more efficient than VM3 (10.2-fold versus 6.2-fold reduction in dsGFP signal and 1.5-fold increased indel frequency) and H1_19 was more effective than H1_19–3 (6.8-fold versus 5.0-fold reduction in dsGFP signal and 1.5-fold increased indel frequency). We therefore selected H1_19, C1, V1 and VM1 for further testing in primary human cells. Of note, one of the R382W-specific nucleases also failed to target the genomic copies of the reporter (Figure 2).

### 3.3. Allele-Specific Disruption of Autosomal Dominant STAT3 in Patient-Derived Human Cells

**Figure 3:**
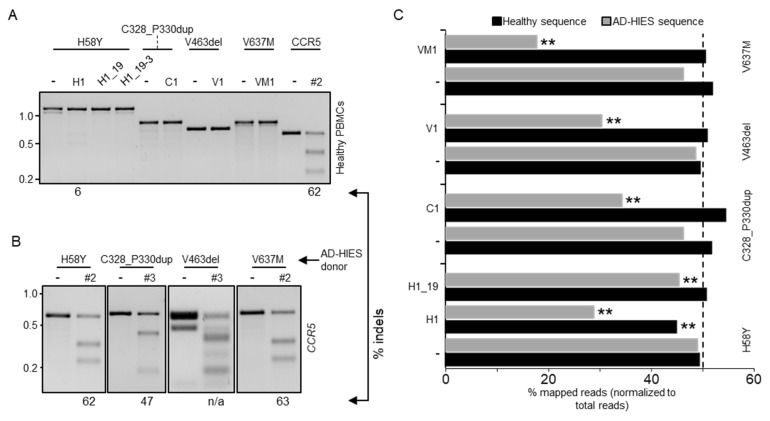
Allele-specificity of selected nucleases in primary human PBMCs. (**A**) Peripheral blood mononuclear cells (PBMCs) from healthy donors were activated to expand the lymphocyte fraction. Three days later, activation beads were removed and the cells were nucleofected with the indicated nuclease or left untreated (-). The presence of indel mutations in the amplicon encompassing the nuclease target site (top) was assessed 10 days later via the T7E1 assay. The percentage of cleaved product is indicated below each panel. (**B**) PBMCs isolated from different AD-HIES patients with heterozygous mutations (indicated on top) were nucleofected with the indicated *CCR5*-specific nuclease (#2 or #3). Nuclease activity was determined via the T7E1 assay. (**C**) Three weeks after nucleofection, genomic DNA was extracted and PCR amplicons encompassing the nuclease target loci in the respective patient-derived PBMCs (left) were subjected to next generation sequencing. The histogram shows the fraction of reads mapped either to the healthy or the AD-HIES alleles. The dashed line indicates the expected fraction of each allele (i.e., 50%). The CRISPResso2 Compare function was used to assess if healthy and AD-HIES alleles were significantly altered when the cells received the indicated nuclease (** *p* < 0.01).

Since the selected nucleases were specific for the mutated *STAT3* alleles, their cognate target sites could only be found in patient-derived cells. Before approaching this source of limited cells, we sought to establish the most efficient conditions to achieve high gene disruption frequencies in human PBMCs. As a control, we used a *CCR5*-specific nuclease (CCR5#2) that was previously established in our laboratory [25]. In addition, we tested the selected *STAT3* allele-specific nucleases for disruption of the non-mutated *STAT3* gene in cells derived from an healthy donor. We activated the PBMCs with beads coupled with anti-CD2, -CD3 or -CD28 antibodies to expand the lymphocyte fraction. The beads were removed from the medium one day prior to nucleofection of the cells with the indicated RNPs. The indel frequency was determined ten days later via the T7E1 assay (Figure 3A). The *CCR5*-specific nuclease efficiently induced indel mutations at its intended target site with frequencies approaching 65%, which is in line with our previous report. Importantly, the *STAT3* allele-specific nucleases did not introduce indel mutation on the healthy *STAT3* allele, while the non-selective H1 nuclease retained its ability to target the *STAT3* gene in primary human cells, introducing indels with a frequency of about 6%. CRISPR-Cas nucleases including the *CCR5*-targeting nuclease as a control were then transferred into PBMCs from STAT3-HIES patients harboring any of the four selected *STAT3* mutations. Since the patient-derived cells expanded more slowly than the healthy donor cells, we measured the indel frequency between days 7 and 14 post-nucleofection via the T7E1 assay (Figure 3B). Indel deposition at the *CCR5* target ranged from 40% to 60% in the patient-derived cells, respectively (Figure 3B). Then, we measured the ability of each of the selected allele-specific nuclease (H1, H1_19, C1, V1, and VM1) to target the mutated *STAT3* allele by next generation targeted amplicon sequencing (NGS).

All of the selected allele-specific nucleases were effective in introducing indel mutations in their respective target sites, with frequencies ranging from 30% to 60% (Figure 3C). Importantly, non-edited healthy *STAT3* alleles represented about 50% of the total NGS reads. As the patients were heterozygous for the *STAT3* mutations, we therefore concluded that all of the tested nucleases were highly selective and able to discriminate between the healthy and mutant *STAT3* alleles. As expected, the non-selective H1 nuclease reduced the count of non-edited normal alleles by about 20%, which is in line with the results achieved with the reporter systems.

### 3.4. Disruption of the Mutated DN-STAT3 Alleles Does Not Rescue STAT3 Signaling

**Figure 4:**
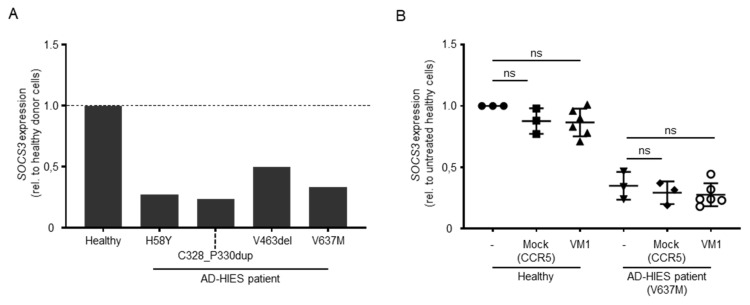
Effect of AD-HIES allele inactivation on STAT3 signaling. (**A**) The histogram shows the expression levels of *SOCS3* (normalized to *HPRT* levels) in the indicated PBMC samples. Data shown are computed as the ratio between *SOCS3* expression levels in the indicated sample and values measured in cells derived from a healthy donor. (**B**) The effect of AD-HIES allele inactivation on the *SOCS3* expression levels was measured via droplet digital PCR in samples receiving the indicated nuclease or left untreated (-). Data shown were computed as the ratio between the *SOCS3* expression levels in the indicated sample and values measured in cells derived from untreated healthy donor cells. Statistically significant differences compared to untreated cells were calculated with one-way ANOVA followed by Dunnett’s multiple comparisons test. ns: non-significant difference.

Previous reports have shown a direct transcriptional upregulation of *SOCS3*, *BATF*, *BCL6*, *FOSL2*, *IKZF2*, *IRF4*, *RORA,* and *SMAD7* upon activation of STAT3 signaling [26]. Conversely, the reduction of STAT3 downstream target gene expression has been reported in cells isolated from AD-HIES patients, particularly for SOCS3. We reasoned that the inactivation of the DN-STAT3 allele would result in an increased formation of functional STAT3 homodimers that would exert their function in the transcriptional activation of target genes. To assess which of the STAT3 transcriptional targets responded to the activation of STAT3 signaling in our in vitro culture conditions, we activated PBMCs from healthy donors and expanded them in vitro in the presence of IL-2. Since this cytokine negatively affects STAT3 signaling [27], we cultured the cells for an additional day in its absence prior to activating the STAT3 pathway by culturing the cells for 1 h in the presence of IL-21 [28]. We then analyzed the expression levels of the above-mentioned eight STAT3 target genes via droplet digital PCR. Under these conditions, three genes were upregulated (i.e., *SOCS3*, *BATF*, and *IRF4* (Figure S3A)). To validate these results in cells that lack STAT3 expression, we artificially inactivated the *STAT3* gene in healthy donor PBMCs using a CRISPR-Cas9 nuclease targeted to exon 21 (Table 1). We achieved indels in up to 57% of target alleles, as measured via the T7E1 assay (Figure S3B). This genetic disruption was associated with a marked decrease in the STAT3 protein levels, as shown by Western blot analysis (Figure S3B). As expected, all three selected STAT3 transcriptional targets were downregulated upon *STAT3* inactivation (Figure S3C). Since *SOCS3* showed the highest reduction in gene expression levels (i.e., 2.3-fold reduction compared to the healthy control cells receiving an unrelated nuclease), we chose to use this gene to monitor for any effects of mutated *STAT3* allele disruption in the AD-HIES patient-derived cells. We first validated reduced *SOCS3* upregulation in patient-derived cells, which ranged between 2- and 4-fold compared to healthy donor cells (Figure 4A). Although allele-specific disruption of the V637M mutation harboring *STAT3* allele was efficacious in patient PBMCs (VM1, Figure 3C), we did not detect the rescue of *SOCS3* activation in VM1-edited patient PBMCs upon stimulation with IL-21 (Figure 4B).

## 4. Discussion

Transplantation of hematopoietic stem cells of allogeneic source has shown unquestioned success for the treatment of multiple human conditions of the blood and immune system including inherited disorders. However, the intrinsic risks of this procedure require careful evaluation of the patient background, and it is often challenging for the physician to decide whether HSCT is a reasonable solution unless it represents the patient’s last resort. The use of autologous stem cells for transplantation represents a valuable alternative as it would mitigate many of the risks associated with allogeneic HSCT such as graft-versus-host disease as a result of incomplete histocompatibility between the cell donor and the receiving patient. However, this approach can be pursued only after correction of the monogenetic defect underlying the disease. First, evidence that CRISPR-Cas9-based therapeutics can be used in humans [29] has opened new avenues for the development of further strategies for the modification of transplantable cells of autologous source. In the context of STAT3-HIES patients, strategies that eliminate the underlying genetic defect in *STAT3* might represent a valuable therapeutic opportunity. However, genome editing strategies to precisely correct a genetic mutation such as those resulting in STAT3-HIES rely on the homology-directed repair (HDR) mechanism. This DNA repair pathway is generally inefficient in mammalian cells and as a consequence, therapeutic genome editing has mostly relied on NHEJ-based gene disruption strategies [16,30].

Taking advantage of the high specificity of the CRIPSR-Cas9 system, we explored this concept in order to selectively disrupt the mutant *STAT3* allele in patient-derived cells. We assumed that the remaining healthy allele would be sufficient to ameliorate the disease in patient cells, similarly to what has been observed in mouse [14]. To establish a strategy that would be applicable to the majority of STAT3-HIES patients, we selected five common mutations found in our local patient cohort. To help the nucleases discriminate between the mutant and the healthy *STAT3* alleles, we explored multiple strategies to destabilize CRISPR-Cas9 binding to the healthy allele. In particular, we used truncated gRNAs as well as the addition of mismatches and achieved allele-specificity for four out of five selected DN-STAT3 mutations. For one mutation (i.e., c.1144C > T (R382W)), we experienced low targeting efficiency for all of the designed nucleases. Since low activity was observed for both the episomal reporter and the genomic alleles while the nuclease was efficiently cleaving the target site in vitro, we reasoned that the epigenetic context was not the cause of the lack of activity. Possibly, the affinity of the CRISPR-Cas9 nuclease for its intended target site was too low for efficient cleavage in cells in the context of a complex genome, but sufficient for in vitro activity on a relatively short DNA fragment. Further tests are necessary to clarify the reason behind the failure in targeting the R382W (c.1144C > T) mutation. Such experiments could provide novel insights on how to select DNA sequences for efficient editing.

Importantly, the nucleases selected from the reporter assays retained their allele selectivity when applied to patient-derived cells. NGS results confirmed the absence of indel formation at the healthy allele for all selected allele-specific CRISPR-Cas9 nucleases, whereas 25% to 62% of the mutated *DN-STAT3* alleles harbored indels. Interestingly, we achieved the most efficient allele-discrimination with CRISPR-Cas9 nucleases that were not destabilized. In contrast, CRISPR-Cas9 complexes with truncated gRNAs were effective in the reporter cell lines, but almost completely lost their editing capacity in primary patient cells (Figure 3C). These findings highlight the limits of using surrogate reporter cell line for the selection of efficient designer nucleases and suggest that it is preferable to use the ultimate target cell type (i.e., healthy donor primary cells) for this purpose, whenever possible.

Despite the high efficiency in disrupting the mutant *STAT3* alleles, and hence causing a state of STAT3 haploinsufficiency in the genetically corrected cells, we failed to achieve a measurable benefit in restoring STAT3 signaling, indicating that insufficiency of STAT3 is likewise pathogenic. The expression levels of *SOCS3*, a direct transcriptional target of STAT3 signaling, were indistinguishable from those measured in non-edited cells. The reasons behind this failure might be multifarious. On one hand, haploinsufficiency might result in the inability to rescue the defect in the presence of a single healthy *STAT3* allele [31]. On the other hand, since negative dominance has been confirmed for many of the *STAT3* pathogenic variance known so far [7], we tended to accept that the consequence of indel formation at the mutation site might result in novel missense, non-sense, or frameshift STAT3 variants with negative dominance. If non-sense mediated decay (NMD) of the resulting transcripts harboring indel mutations is inefficient, translation of the resulting STAT3 variants may contribute to the dysfunction in STAT3 signaling. However, again, such variants more likely lead to a state of functional haploinsufficiency, indicating that 50% of healthy *STAT3* alleles are still not enough to secure normal STAT3 signaling. Such possibilities have not been highlighted in a recent study aimed at the inactivation of a mutated *KRT5* allele, causing epidermolysis bullosa simplex [32]. While the disease background might play a critical role in the outcome of allele-specific disruption, characterizing the predominantly expressed indel variants upon genome editing and experimentally testing their mechanism of action might contribute to confirm their contribution to the observed outcome. Certainly, our results underline important differences between the human and the mouse immune system and suggest that while a single *STAT3* allele deletion might be tolerated in mouse [14], this condition is not sufficient to restore signaling in human cells that are apparently more vulnerable to STAT3 levels.

In general, the presented results have important consequences for the genome editing field. Our findings suggest that using designer nucleases to target protein-coding regions with the goal of inactivating a mutant allele with dominant-negative effects entails a non-trivial risk of failure. First, haploinsufficiency needs to be excluded as being disease-causing. Furthermore, and maybe even more importantly, when pursuing HDR-mediated precision editing to correct a disease-underlying mutation, the simultaneous generation of alleles harboring indel mutations in the target cell population might represent a risk that has to be carefully evaluated. In conclusion, our results demonstrate that CRISPR-Cas9 nucleases offer the opportunity of allele-specific editing with single base resolution. However, when targeting protein-coding regions, imprecise cellular DNA repair pathways may represent a concern. The use of editing strategies that avoid the insertion of DNA double strand breaks such as base editing [33] or epigenome editing [34], represent valuable alternatives for clinical translation.

## Figures and Tables

**Table 1:** Designer nucleases target sites.

Target Allele	ID	Protospacer+PAM (5’-->3’) ^a^	Notes
*CCR5*	mock/#2	CAATGTGTCAACTCTTGACA**GGG**	Full-length
	#3	ATTTCCAAAGTCCCACTGGG**CGG**	
*STAT3*	S_KO	TAAGACCCAGATCCAGTCCG**TGG**	
*H58Y*	H1	GAGATTATAAAACACCAAAG**NGG**	
	H2	CAGGAGATTATAAAACACCA**NAG**	
*C328_P330dup*	C1	ATGGGCATGCAGGGCATGCA**NGG**	
	C2	CATGGGCATGCAGGGCATGC**NGG**	
*R382W*	R2	ATCCTGGAAATTTAACATTC**NGG**	
	R3	TTAAATTTCCAGGATCCTCT**NAG**	
*V463del*	V1	CAGATGTTGGAGATCACAAC**NGG**	
*V637M*	VM1	TAAGACCCAGATCCAGTCCA**NGG**	
	VM3	TTGTGTATGGTTCCATGGAC**NGG**	
*H58Y*	H1_19	GATTATAAAACACCAAAG**NGG**	Truncated
	H2_19	GGAGATTATAAAACACCA**NAG**	
*V463del*	V1_19	GATGTTGGAGATCACAAC**NGG**	
*V637M*	VM1_17	GACCCAGATCCAGTCCA**NGG**	
*H58Y*	H1_19+1	GATTAAAAAACACCAAAG**NGG**	truncated and mismatched
	H1_19+2	GATTTTAAAACACCAAAG**NGG**	
	H1_19+3	GATAATAAAACACCAAAG**NGG**	
	H1_19-1	GATTATATAACACCAAAG**NGG**	
	H1_19-2	GATTATAATACACCAAAG**NGG**	
	H1_19-3	GATTATAAATCACCAAAG**NGG**	
*R382W*	R1_19	GCCCAGAATGTTAAATTTC**NAG**	
	R2_19+4	GTCCTGGAAATTTAACATTC**NGG**	
	R2_18+3	GCCTGGAAATTTAACATTC**NGG**	
	R2_17+2	GCTGGAAATTTAACATTC**NGG**	
	R3_19+10	GTAAATTTCCAGGATCCTCT**NAG**	
	R3_18+9	GAAATTTCCAGGATCCTCT**NAG**	
	R3_17+8	GAATTTCCAGGATCCTCT**NAG**	

^a^ Protospacer adjacent motif (PAM) is indicated in bold letters.

## Data Availability

The data presented in this study are available on request from the corresponding author.

## Supplementary Materials

The following are available online at https://www.mdpi.com/article/10.3390/genes13101912/s1, Figure S1: Measurement of transfection efficiency, Figure S2: Cleavage ability of selected nucleases in an in vitro cleavage assay, Figure S3: Functional measurement of STAT3 signaling, Table S1: Sequences of primers used in this study, Table S2: Copy number estimation.
