# Supplementary material for: Allele-Specific Disruption of a Common STAT3 Autosomal Dominant Allele Is Not Sufficient to Restore Downstream Signaling in Patient-Derived T Cells
Source: Genes (Basel). 2022 Oct 20;13(10):1912. doi: 10.3390/genes13101912 (PMC9601366; doi:10.3390/genes13101912)
Supplement: Supplementary file 1 [file genes-13-01912-s001.zip › genes-1914043-supplementary.pdf]

Supplementary Material

Allele-specific disruption of a common *STAT3* autosomal dominant allele is not sufficient to restore downstream signaling in patient-derived T cells

Saskia König, Markus Hildenbeutel, Manfred Fliegauf, Bodo Grimbacher, Tatjana I. Cornu, Toni Cathomen, and Claudio Mussolino

**Supplementary Figure S1**	Transfection efficiency
**Supplementary Figure S2**	*In vitro* cleavage assay
**Supplementary Figure S3**	Functional measurement of STAT3 signaling
**Supplementary Table S1**	Sequences of primers used in this study
**Supplementary Table S2**	Copy number estimation

Koenig et al – Supplementary Figure S1

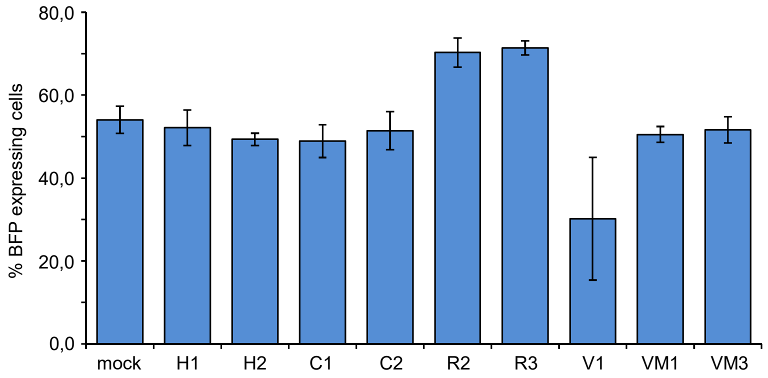

Supplementary Figure S1. Transfection efficiency. The histogram shows the percentage of BFP-positive cells, as measured by flow cytometry 24 hours after transfection. Experiments were performed at least in triplicates

Koenig et al – Supplementary Figure S2

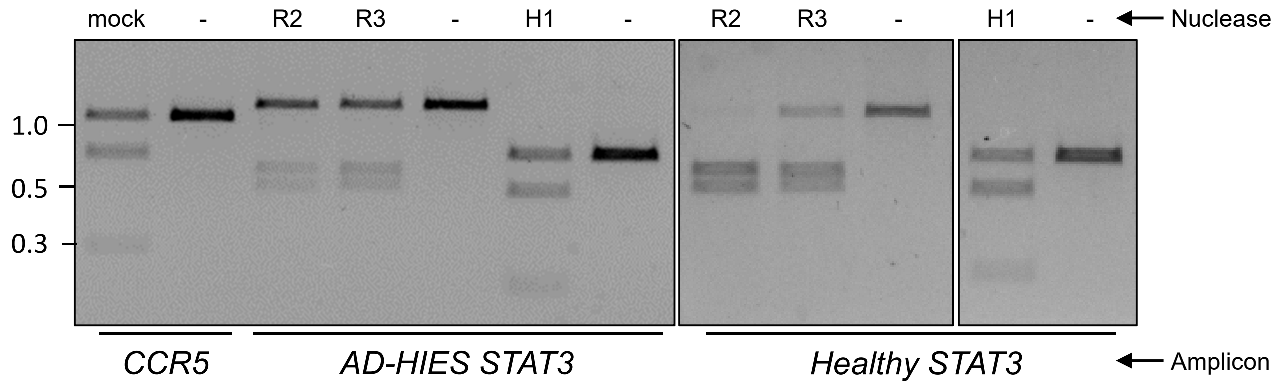

Supplementary Figure S2. I*n vitro* cleavage assay. The Cas9 protein and the synthetic sgRNA indicated on top were mixed with the corresponding DNA fragment indicated below the image. The components were incubated at 37°C for 90 minutes before reaction was stopped by the addition of RNase A and Proteinase K. A nuclease targeted to the CCR5 gene was included as positive control. -: negative control in which both the Cas9 protein and the corresponding sgRNAs were omitted. DNA-ladder (in kb) is indicated on the left.

Koenig et al – Supplementary Figure S3

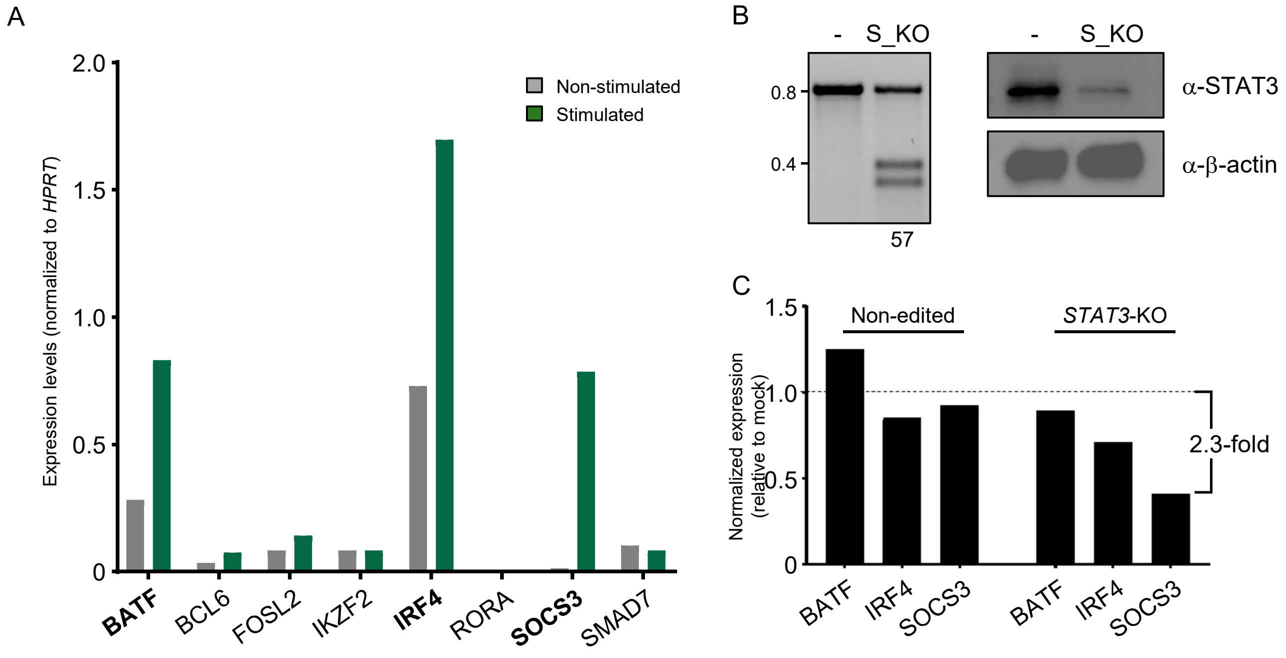

Supplementary Figure S3. Functional measurement of STAT3 signaling. (**A**) PBMCs of healthy donors were stimulated with IL-21 for 1 hour prior measuring the expression levels of STAT3 downstream transcriptional targets via droplet digital PCR. The targets that respond to STAT3 activation in the culture condition tested were selected for subsequent experiments (bold). (**B**) A CRISPR-Cas9 targeted to exon 21 of the *STAT3* gene (S_KO) was used to artificially knock out STAT3 in healthy PBMCs. Efficacy of target gene disruption was assessed at the genomic level, via T7E1 assay (left), or protein level, via western blotting (right). (**C**) The expression levels of selected STAT3 transcriptional targets were measured via droplet digital PCR upon stimulation with IL-21 for 1 hour. The histogram shows the expression level of each gene, normalized to HPRT1, in the sample indicated (top) relative to a sample receiving an unrelated *CCR5*-specific CRISPR-Cas9 used as a negative control (mock).

Supplementary Table S1. Sequences of primers used in this study

Sequence (5'--> 3')[a, b]	Purpose	ID
acaccGAGATTATaAAACACCAAAGg	Oligo-cloning	sgRNA H58Y (H1)
aaaacCTTTGGTGTTTtATAATCTCg	Oligo-cloning	
acaccGATTATaAAACACCAAAGg	Oligo-cloning	sgRNA H58Y (H1_19)
aaaacCTTTGGTGTTTtATAATCg	Oligo-cloning	
acaccGATTATaTAACACCAAAGg	Oligo-cloning	sgRNA H58Y (H1_19-1)
aaaacCTTTGGTGTTAtATAATCg	Oligo-cloning	
acaccGATTATaATACACCAAAGg	Oligo-cloning	sgRNA H58Y (H1_19-2)
aaaacCTTTGGTGTATtATAATCg	Oligo-cloning	
acaccGATTATaAATCACCAAAGg	Oligo-cloning	sgRNA H58Y (H1_19-3)
aaaacCTTTGGTGATTtATAATCg	Oligo-cloning	
acaccGATTAAaAAACACCAAAGg	Oligo-cloning	sgRNA H58Y (H1_19+1)
aaaacCTTTGGTGTTTtTTAATCg	Oligo-cloning	
acaccGATTTTaAAACACCAAAGg	Oligo-cloning	sgRNA H58Y (H1_19+2)
aaaacCTTTGGTGTTTtAAAATCg	Oligo-cloning	
acaccGATAATaAAACACCAAAGg	Oligo-cloning	sgRNA H58Y (H1_19+3)
aaaacCTTTGGTGTTTtATTATCg	Oligo-cloning	
acaccCAGGAGATTATaAAACACCAg	Oligo-cloning	sgRNA H58Y (H2)
aaaacTGGTGTTTtATAATCTCCTGg	Oligo-cloning	
acaccGGAGATTATaAAACACCAg	Oligo-cloning	sgRNA H58Y (H2_19)
aaaacTGGTGTTTtATAATCTCCg	Oligo-cloning	
acaccATGGGCATGCAGGGCATGCAg	Oligo-cloning	sgRNA C3dup (C1)
aaaacTGCATGCCCTGCATGCCCATg	Oligo-cloning	
acaccCATGGGCATGCAGGGCATGCg	Oligo-cloning	sgRNA C3dup (C2)
aaaacGCATGCCCTGCATGCCCATGg	Oligo-cloning	
acaccCAGATGTTGGAGATCACAACg	Oligo-cloning	sgRNA V4delta (V1)
aaaacGTTGTGATCTCCAACATCTGg	Oligo-cloning	
acaccGATGTTGGAGATCACAACg	Oligo-cloning	sgRNA V4delta (V2)
aaaacGTTGTGATCTCCAACATCg	Oligo-cloning	
acaccTAAGACCCAGATCCAGTCCAg	Oligo-cloning	sgRNA V637M (VM1)
aaaacTGGACTGGATCTGGGTCTTAg	Oligo-cloning	
acaccGGTTCCATGGACTGGATCg	Oligo-cloning	sgRNA V637M (VM3)
aaaacGATCCAGTCCATGGAACCg	Oligo-cloning	
acaccGCCCAGAATGTTAAATTTCg	Oligo-cloning	sgRNA R382W (R1_19)
aaaacGAAATTTAACATTCTGGGCg	Oligo-cloning	
acaccATCCTGGAAATTTAACATTCg	Oligo-cloning	sgRNA R382W (R2)
aaaacGAATGTTAAATTTCCAGGATg	Oligo-cloning	
acaccGTCCTGGAAATTTAACATTCg	Oligo-cloning	sgRNA R382W (R2_19)
aaaacGAATGTTAAATTTCCAGGACg	Oligo-cloning	
acaccGCCTGGAAATTTAACATTCg	Oligo-cloning	sgRNA R382W (R2_18)
aaaacGAATGTTAAATTTCCAGGCg	Oligo-cloning	
acaccGCTGGAAATTTAACATTCg	Oligo-cloning	sgRNA R382W (R2_17)
aaaacGAATGTTAAATTTCCAGCg	Oligo-cloning	
acaccTTAAATTTCCAGGATCCTCTg	Oligo-cloning	sgRNA R382W (R3)
aaaacAGAGGATCCTGGAAATTTAAg	Oligo-cloning	
acaccGTAAATTTCCAGGATCCTCTg	Oligo-cloning	sgRNA R382W (R3_19)
aaaacAGAGGATCCTGGAAATTTACg	Oligo-cloning	
acaccGAAATTTCCAGGATCCTCTg	Oligo-cloning	sgRNA R382W (R3_18)
aaaacAGAGGATCCTGGAAATTTCg	Oligo-cloning	
acaccGAATTTCCAGGATCCTCTg	Oligo-cloning	[illegible]

aaaacAGAGGATCCTGGAAATTCg	Oligo-cloning	sgRNA R382W (R3_17)
CCCTGGCGTCGTGATTAG	ddPCR	HPRT1-4406
CATGAGGAATAAACACCCTTTCC	ddPCR	HPRT1-4407
CTTCGATTCGGGACCAGCC	ddPCR	SOCS3-4428
CGGAGCCAGCGTGGATC	ddPCR	SOCS3-4429
ATGCTTGTGCCCCACCTG	ddPCR	IRF4-4424
GTGCAGCCGGCAGTCTG	ddPCR	IRF4-4425
CCCTGGCAAACAGGACTC	ddPCR	BATF-4416
GGTCTTCGCTCTCCAGGTG	ddPCR	BATF-4417
GATGAATGGGTTATAGCATCAGGTTTGCTTTG	NGS	H58Y-4024
GTCCTGTTTCTCCTTGTCCTCAGT	NGS	H58Y-4025
TTAGCATCCTTGTCCCTTTCCCTC	NGS	C3dup-4205
AGAATGACCCTGGCCACCAAC	NGS	C3dup-4206
ACTCCTCGCCTAGAGTTGGCA	NGS	V4delta-4207
TACTAGGTACCCCTAAGTCGCAAG	NGS	V4delta-4208
CAGGGTGTTCAGGGTCTCAACACT	NGS	V637M-4203
ACTTTCCGAATGCCTCCTCCTTG	NGS	V637M-4204

[a] *Overhangs are indicated in small letters while the sgRNA sequence in capital letters.*

[b] *ODNs annealing on the reverse DNA strand are indicated in italics.*

Supplementary Table S2. Copy number estimation

Cell type	Gene ID	Concentration (copies/µl)	Genomic copies (rel. to PTPB2 in PBMCs)
PBMC	*PTBP2*	295,0	2,0
	Reporter	10,3	0,1
	STAT3	354,0	2,4
HEK293T	*PTBP2*	436,0	3,0
	Reporter	8,6	0,1
	STAT3	519,0	3,5
HEK293Tmut (clone #2)	*PTBP2*	394,0	2,7
	Reporter	396,0	2,7
	STAT3	531,0	3,6
HEK293Tmut (clone #7)	*PTBP2*	444,0	3,0
	Reporter	229,0	1,6
	STAT3	577,0	3,9
